# Dyspnea after discharge from hospital due to pulmonary vein thrombosis after video-assisted left upper lobectomy: a case report

**DOI:** 10.1186/s40981-022-00567-8

**Published:** 2022-09-30

**Authors:** Ruiji Kubo, Takuo Hoshi, Akae Shu, Yuichiro Yamasaki

**Affiliations:** 1 Department of Anesthesiology and Critical Care Medicine, Ibaraki Prefectural Central Hospital, Koibuchi 6528, Kasama, Ibaraki 309-1793 Japan; 2 Department of Anesthesiology and Critical Care Medicine, Ibaraki Clinical and Training Center, University of Tsukuba Hospital, Koibuchi 6528, Kasama, Ibaraki 309-1793 Japan

**Keywords:** Thoracic surgery, Postoperative analgesia, Postoperative complication, Thromboembolism

## Abstract

**Background:**

Thrombus formation at the pulmonary vein transection site is more common in left upper lobectomy than other lobectomies. We report a case of dyspnea and contrast-enhanced computed tomography (CT) findings of pulmonary vein thrombosis after left upper lobectomy.

**Case presentation:**

A 68-year-old man with left lung cancer underwent video-assisted thoracoscopic left upper lobectomy under general anesthesia with thoracic epidural analgesia. He had no postoperative complications and was discharged home on the 5th day postoperatively. He visited the outpatient clinic at 8 days after surgery because of dyspnea and underwent contrast-enhanced CT, which revealed a thrombus at the resected edge of the left upper pulmonary vein. Anticoagulation therapy was started. Thereafter, the thrombus shrank, and the patient’s dyspnea improved.

**Conclusions:**

Left upper lobe resection is particularly associated with pulmonary venous thrombosis, and dyspnea due to pulmonary venous thrombus may develop late after surgery. Postoperative management methods such as anticoagulation and postoperative pain management should be reexamined.

## Background

Video-assisted thoracoscopic surgery (VATS) is often considered the standard technique for treating lung cancer, and epidural analgesia is often used for intraoperative and postoperative pain relief. Thrombus formation at the pulmonary vein transection site, a risk factor for postoperative cerebral thromboembolism, is more common in left upper lobectomy than other lobectomies [1]. We report a case of pulmonary vein thrombosis after left upper lobectomy. The patient underwent left upper lobectomy via VATS under general anesthesia with epidural analgesia and was discharged on the 5th postoperative day. However, 8 days postoperatively, he experienced dyspnea and was diagnosed with pulmonary vein thrombosis. Written patient consent was obtained for the publication of this report, which was prepared according to the CARE guidelines.

## Case presentation

**Fig. 1:**
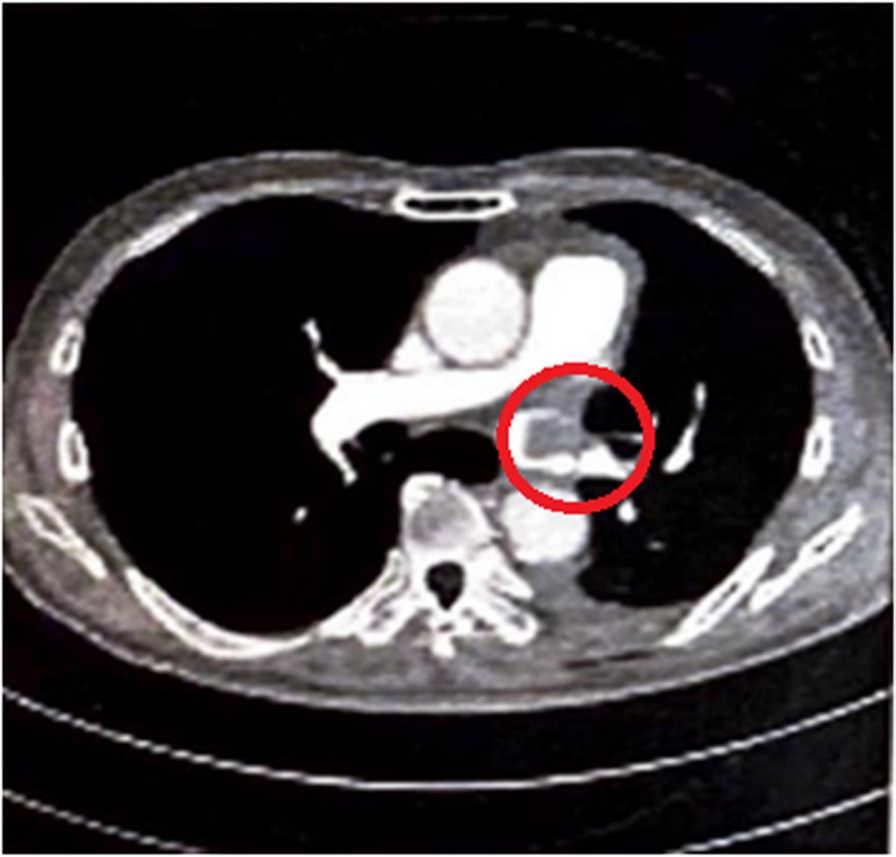
Contrast-enhanced chest computed tomography image. The circle indicates the pulmonary vein thrombosis

A 68-year-old man (height: 169 cm, weight: 68 kg) who was receiving medical treatment for hypertension and diabetes mellitus was diagnosed with stage 1B adenocarcinoma of the lung based on the findings of investigations including computed tomography (CT) after an abnormal shadow was noted in his left upper lung field on a chest radiograph during a medical checkup. He was scheduled for left upper lobectomy via VATS. Surgery was performed under general anesthesia with thoracic epidural analgesia in the right lateral recumbent position. Before induction of general anesthesia, a thoracic epidural catheter was placed in Th4/5, induction was done with propofol and remifentanil, muscle relaxation was obtained with rocuronium, and double-lumen tube was intubated endotracheally to maintain anesthesia with intermittent lidocaine administration into the epidural space and desflurane inhalation. On the first postoperative day, the patient started eating and getting out of bed, but atrial fibrillation with a heart rate of 120–150 beats/min appeared. A transdermal patch of bisoprolol 4 mg/day was applied, and the heart rate was maintained at 80–100 beats/min. Patient-controlled epidural analgesia provided good pain control with the Prince Henry Hospital pain scale is less than or equal to 2 and was used until the 4th postoperative day. Although atrial fibrillation persisted, the heart rate was controlled well with bisoprolol, and there were no other complications; therefore, the patient was discharged on the 5th postoperative day. On the 7th postoperative day, the patient experienced light-headedness and dyspnea while walking to the bathroom, but the symptoms quickly improved. On the 8th postoperative day, the patient experienced dyspnea and visited our hospital. Contrast-enhanced CT revealed a thrombus (18 mm in diameter) attached to the resected edge of the left pulmonary vein (Fig. 1), although no pulmonary edema was observed, blood flow in the pulmonary veins was greatly impeded. The patient was immediately readmitted to the hospital, and an echocardiogram was obtained, which showed no evidence of right heart overload, heart failure, or hypoxemia. Heparin 18,000 units/day was immediately started. However, the activated partial thromboplastin time did not prolong, even after the dose was increased to 25,000 units/day. Therefore, the patient was switched to 60 mg of edoxaban tosilate hydrate. Sinus rhythm was achieved on the 10th postoperative day, and the patient was discharged on the 24th postoperative day because the thrombus was shrinking, and no dyspnea was observed. The thrombus was confirmed to have disappeared on the 66th postoperative day.

## Discussion

Pulmonary vein thrombosis is a rare but potentially serious condition. Pulmonary neoplasms, complications of lung transplantation, lobectomy, or radiofrequency ablation, fibrosing mediastinitis, and mitral stenosis with a left atrial clot are known etiology causes of pulmonary vein thrombosis [2]. Thrombosis at the pulmonary vein transection site reportedly occurs in 11.7–30.8% of patients undergoing left upper lobectomy [1, 3, 4]. Care must be taken because cerebral infarction is relatively frequently reported after left upper lobectomy [4, 5], and left pneumonectomy and left upper lobectomy are considered independent risk factors for postoperative stroke [6].

The mechanism of pulmonary vein thrombosis is not fully understood, but it is thought to be related to the fact that the pulmonary vein stump tends to be longer after left upper lobectomy than after other lobectomies despite the surgeon’s efforts to shorten it as much as possible [7]. This leads to decreased blood flow in the left atrium and no blood flow from the bifurcation, which increases the likelihood of turbulent flow and stasis [8]. Additionally, intraoperative pulmonary vein transection damages the vascular endothelium, which causes activation of the extrinsic cascade of coagulation [7].

Most patients with pulmonary vein thrombosis are asymptomatic, but pulmonary vein stenosis can cause nonspecific symptoms such as cough, hemoptysis, and dyspnea [8]. Although our patient had no obvious pulmonary edema, blood flow in the pulmonary veins was greatly impeded. Furthermore, since the patient had no other pulmonary or cardiac disease that could have caused dyspnea, we considered pulmonary vein stenosis to be the probable cause of dyspnea.

In a multicenter observational study, most pulmonary vein thrombosis was detected within the first postoperative week [4]. Although it is unclear whether atrial fibrillation leading to blood stasis is a risk factor for thrombus development and there is also uncertainty regarding the thromboembolic risk associated with left upper pulmonary vein thrombus in atrial fibrillation [8], the pulmonary vein thrombus in this case may have begun to form early in the postoperative period.

Anticoagulant drug therapy is considered in patients with pulmonary vein thrombosis, but its use remains controversial due to the risk of postoperative bleeding. Although 95% of patients with pulmonary vein thrombosis are diagnosed within the first week of surgery [4], the optimal time to begin anticoagulation and the optimal duration of treatment remain unclear. Among patients with pulmonary vein thrombus after lung surgery, 68.5% received anticoagulants, and the thrombus resolved in 94.3% of them [4]. Hemorrhagic complications such as epidural hematoma have not been reported, but caution should be exercised when epidural analgesia is used. There are reports of routine systemic heparin administration for 3 days after left upper lobectomy, during which epidural analgesia is replaced with an intercostal nerve block, but the safety of this has not been confirmed [9]. In addition, recently published European guidelines do not recommend epidural analgesia in patients undergoing VATS but recommend the use of a paravertebral block or spinal erector spinae plane block in combination with general anesthesia [10], because epidural analgesia is more invasive intervention and conveys the risks of hypotension, urinary retention, and potential lower limb weakness, which can delay early rehabilitation and resumption of walking.

## Conclusion

We report a case of delayed dyspnea due to pulmonary vein thrombosis on the 8th day after left upper lobectomy via VATS. Left upper lobe resection is particularly associated with pulmonary venous thrombosis, and postoperative management methods such as anticoagulation and postoperative pain management without epidural analgesia may need to be considered.

## Data Availability

Not applicable.

## Abbreviations

VATS: Video-assisted thoracoscopic surgery
CT: Computed tomography
